# Infantile Hypertrophic cardiomyopathy: steps towards an evidence-based approach to genetic testing

**DOI:** 10.1038/s41390-025-04188-6

**Published:** 2025-06-10

**Authors:** Gabrielle Norrish, Juan Pablo Kaski

**Affiliations:** 1 Centre for Paediatric Inherited & Rare Cardiovascular Disease, Institute of Cardiovascular Science, London, UK; 2 Centre for Inherited Cardiovascular Diseases, Great Ormond Street Hospital, London, UK

For decades, hypertrophic cardiomyopathy (HCM) in children was seen as a condition distinct from adult-onset HCM, characterised by severe disease with poor outcomes, and caused largely by inborn errors of metabolism and malformation syndromes. In the last 10 years, however, this notion has been largely debunked, with the demonstration that most cases of childhood-onset HCM beyond infancy (the first year of life) are caused by variants in the same sarcomeric genes that cause adult HCM and with short and medium-term outcomes much more in keeping with those in adult cohorts.^1–3^ This has led to a paradigm change in clinical practice, with the most recent European and North American clinical practice guidelines now recommending genetic testing for all patients with a diagnosis of HCM, regardless of age.^4,5^ Indeed, the overall yield of genetic testing in childhood HCM is higher than in adult populations.^1,6^ Nearly one quarter of HCM diagnosed during childhood presents in infancy^7,8^ and while the underlying aetiology, natural history, and outcomes in this group of patients is extremely variable,^1,7,9^ a substantial minority of infant-onset HCM is also caused by sarcomeric mutations, and it is now recognised that although syndromic disease is more commonly seen in these very young patients, the aetiology of disease is more similar than different to that seen in adulthood.^1,6^ Despite this, the best approach to genetic testing remains unclear in this patient population.

## Genetic basis of infant-onset HCM

The study by Svetlana and colleagues in this issue of the Journal^10^ describes the aetiology, natural history, and outcomes of a single-centre cohort of children presenting with infant-onset HCM, with a particular focus on the yield and results of genetic testing. Their findings corroborate those reported previously for infantile cohorts,^1,7,8^ including a heterogeneous underlying aetiology that includes sarcomeric disease, RASopathy syndromes and inborn errors of metabolism; a variable cardiac phenotype; and similar disease-related complications (e.g. heart failure or sudden death) to patients presenting later in childhood. As in previous reports, the cause of death and mortality rates differed by underlying aetiology; those with metabolic-associated HCM had the lowest survival, providing further support that a focus on determining aetiology is essential for predicting likely disease trajectories and outcomes. A major point of interest is the result of genetic testing in the reported cohort. Previous, larger, multi-centre studies have described the genetic architecture of infantile HCM but have been limited by heterogeneity in both the availability of genetic testing and testing strategies.^1,7,8^ As a single-centre study, a major strength is a consistent and uniform approach to genetic testing within a contemporary timeframe (last 15 years), allowing us to draw some conclusions about the best approach to genetic testing in this age group. Remarkably, over 80% of patients had a likely disease-causing variant identified through genetic testing, providing further evidence that the yield of genetic testing in infantile HCM is at least as high, if not higher, than testing in unselected childhood cohorts. There is, therefore, little question that genetic testing is essential for all patients with a diagnosis of HCM, even in the very young. Furthermore, amongst patients with non-syndromic disease, the genetic make-up of infantile HCM was similar to that described in later childhood. There was a predominance of *MYH7* variants, seen in almost two-thirds of patients, with other genes less commonly involved. This contrasts with adult cohorts in which variants in *MYBPC3* are typically the most prevalent.^11,12^ However, given the small cohort size, it is important to note that although less commonly seen, disease related to other sarcomeric genes, including *MYBCP3*, can also occur in infancy.^6,13^ In adult cohorts, studies have suggested that whole-genome or exome strategies offer only marginal increases in the yield of genetic testing on top of panel testing.^11^ As a result, gene panels comprising only curated genes with sufficient evidence to be disease-causing are frequently used as the first line for genetic testing. In contrast, different approaches to genetic testing have been advocated for early-onset HCM. Given the higher likelihood of syndromic or metabolic disease, some have suggested that initial testing with whole-genome or exome sequencing could be cost-effective and increase the yield of testing.^14^ Interestingly, in this study, most disease-causing variants, including those for syndromic disease, were in genes that would likely be included in contemporary gene panels for HCM, with whole genome sequencing identifying potentially disease-causing variants in almost one quarter, including potentially novel genes. These findings suggest that similar gene panels for infantile, childhood and adult populations may be appropriate as first-line testing, with expansion to whole genome or exome sequencing if initial panel testing is negative. The cost-effectiveness of this approach needs further exploration.

## Gene-elusive HCM and environmental modifiers

Genotype-elusive adult patients have historically been described to be older, less likely to have a family history and more likely to have cardiovascular comorbidities, including hypertension, obesity and diabetes.^15^ Recent studies have suggested that the inheritance pattern in these individuals is more likely to be polygenic in nature, with common benign variants contributing to the likelihood of disease development.^16^ The proportion of gene-elusive individuals in the study by Svetlana et al. was lower than seen in adult cohorts. This is perhaps not unexpected, given the absence of other cardiovascular comorbidities known to be associated with the development of a phenotype later in life. It is currently unknown if the phenotype and natural history of this group of young patients differ from adult genotype elusive patients and if a similar polygenic inheritance pattern is important for disease expression. Well-designed, longitudinal multi-centre studies would be required to answer this question, given the rarity of infantile-onset disease and high yield of genetic testing for this patient group.

In recent years, there has been an increased recognition and awareness of the impact of lifestyle and environment on disease phenotype for adults with sarcomeric and gene-elusive HCM.^15–17^ To date, there has been little discussion about the modifying effect of such factors in childhood disease. It is well-described that maternal gestational diabetes is associated with infantile left ventricular hypertrophy, a finding that typically resolves spontaneously during follow-up.^18^ Interestingly, Svetlana et al. observed that gestational diabetes was seen commonly in the mothers of infants with variants in Z-disc proteins. Such genes are not typically described in early-onset disease, which could suggest that the presence of additional environmental factors could have led to earlier than expected disease expression. The progression of phenotype during early childhood and into adulthood was not described in this study, but it would be interesting to know if the hypertrophy resolved or persisted with the removal of the environmental ‘second hit’. Future studies exploring the role of environment and phenotype development during childhood could provide future opportunities for disease modification.
